# Prevalence of rifampicin-resistant tuberculosis and its associated factors among patients with tuberculosis at Galkayo General Hospital, Puntland, Somalia: a facility-based cross-sectional study

**DOI:** 10.11604/pamj.2026.54.34.51751

**Published:** 2026-06-08

**Authors:** Hawo Mohamud, Kirubel Minsamo Mishore, Bisrat Hagos, Farah Said Barre, Asia Mohammed Ahmed, Tewodros Assefa, Abera Jambo, Assefa Tola, Abraham Nigussie Mekuria

**Affiliations:** 1 Department of Pharmacy, Puntland State University, Puntland, Somalia,; 2 Department of Clinical Pharmacy, School of Pharmacy, College of Health and Medical Science, Haramaya University, Harar, Ethiopia,; 3 Department of Social and Administrative Pharmacy, School of Pharmacy, College of Health and Medical Science, Haramaya University, Harar, Ethiopia,; 4 Department of National Tuberculosis Program, Ministry of Health, Puntland, Somalia,; 5 Department of Epidemiology, School of Public Health, College of Health and Medical Sciences, East Africa University, Qardho, Somalia,; 6 Department of Epidemiology and Biostatistics, School of Public Health, College of Health and Medical Science, Haramaya University, Harar, Ethiopia,; 7 Department of Pharmacology, School of Pharmacy, College of Health and Medical Science, Haramaya University, Harar, Ethiopia

**Keywords:** Mycobacterium tuberculosis, rifampicin-resistant tuberculosis, associated factors, Puntland, Somalia

## Abstract

**Introduction:**

drug-resistant tuberculosis (TB) remains a major public health challenge globally. Monitoring its prevalence and associated factors is essential for effective control strategies. The current study aimed to assess the burden of rifampicin-resistant TB (RR-TB) and its associated factors among patients with TB at Galkayo General Hospital, Puntland, Somalia.

**Methods:**

a facility-based cross-sectional study was conducted using medical records of patients with TB registered from January 2020 to December 2024. Of 422 randomly reviewed records, 410 with complete data were included in the analysis. Descriptive statistics were used to summarize patient details. Univariate and multivariable logistic regression analyses were used to identify variables associated with RR-TB. Adjusted odds ratios (aOR) with 95% confidence intervals (CIs) were reported. A p-value <.05 was deemed statistically significant.

**Results:**

the mean age of participants was 29.9 ± 20.6 years, and 64.6% were male. The prevalence of RR-TB was 9.02% (37/410: 95% CI 6.43%-12.22%). In multivariable logistic analysis, having a history of close contact with a patient with TB (aOR: 2.43, 95% CI 1.13-5.21; P=.02), previous history of TB (aOR: 3.63, 95% CI 1.72-7.67; P<.001), and treatment failure (aOR: 9.03, 95% CI 2.03-40.12; P<.01) were strongly associated with RR-TB.

**Conclusion:**

rifampicin-resistant tuberculosis represents a substantial burden among patients with TB in Galkayo General Hospital. Close contact with TB cases, previous TB history, and treatment failure were significant predictors of RR-TB.

## Introduction

Tuberculosis (TB) continues to pose a major public health challenge worldwide despite the availability of effective prevention and treatment strategies [1]. According to the World Health Organization (WHO) Global TB Report 2024, approximately 10.7 million individuals developed TB and 1.23 million deaths were recorded in 2024 [2]. The growing emergence of drug-resistant TB poses a major challenge to global TB control efforts [3]. Rifampicin-resistant TB (RR-TB), defined as resistance to rifampicin with or without resistance to other 1^st^-line drugs, is particularly important. This is because RR serves as a consistent surrogate indicator for multidrug-resistant TB (MDR-TB) [4]. Multidrug-resistant TB is linked with prolonged treatment, higher healthcare costs, poorer treatment outcomes, and increased mortality compared with drug-susceptible TB [5,6]. Globally, approximately 390,000 new cases of MDR/RR-TB were estimated to occur in 2024, with the highest burden reported in low- and middle-income countries (LMICs) [2]. Multidrug-resistant development is often linked to previous TB treatment, poor adherence, inadequate regimens, and community transmission of resistant strains [7,8].

Hence, early detection and identification of these risk factors are essential for strengthening TB control programs and reducing the transmission of MDR/RR-TB strains [9]. In high TB-burden settings, including countries in sub-Saharan Africa, the transmission of MDR/RR-TB strains has become an increasing concern [10]. Somalia remained among the 30 high MDR/RR-TB burden countries in 2024 [2], partly due to limited diagnostic capacity, weak health systems, and ongoing humanitarian crises [11]. Additionally, large populations of internally displaced persons and refugees, overcrowding, and limited healthcare access increase the risk of TB transmission and MDR/RR-TB emergence [12,13]. Recent facility-based studies conducted by Mohamed *et al*. [13] and Ali *et al*. [14] in Somalia reported MDR/RR-TB prevalence rates of 13.3% and 35%, respectively, among TB patients attending these facilities. However, these findings are limited to a few selected facilities and may not adequately represent the burden of RR-TB in other hospitals and regions of the country. The scarcity of data from different geographic areas and health facilities limits a comprehensive understanding of the magnitude and determinants of RR-TB in Somalia. This study aimed to assess the prevalence of RR-TB and its associated factors among patients with TB at Galkayo Hospital, Puntland, Somalia.

## Methods

**Study design and setting:** a facility-based retrospective cross-sectional study was conducted from March 1^st^ to April 30^th^, 2025, at Galkayo General Hospital, Puntland, Somalia. The hospital is a regional referral facility that provides comprehensive health services to a large catchment population, including internally displaced persons and residents from neighbouring regions [15].

**Study population and sample size:** all patients with TB registered at Galkayo General Hospital between January 1^st^, 2020, and December 31^st^, 2024, constituted the source population. The study population comprised eligible patients with TB whose records were reviewed during the data collection period from March 1^st^ to April 30^th^, 2025. Incomplete records (e.g., missing drug susceptibility test results or diagnosis details) were excluded from the analysis. A single population proportion formula was used to determine the sample size, assuming a 50% prevalence of RR-TB (p=0.5) due to the absence of prior studies in the study setting. A 95% confidence level (z=1.96) and a 5% margin of error (d=0.05) were considered. Accordingly, 422 medical records were randomly selected and proportionally allocated for each year.

**Data collection:** patients diagnosed with TB from January 1^st^, 2020, to December 31^st^, 2024, were identified from the TB registration book. Corresponding medical records were retrieved, and data were extracted by trained data collectors using a pretested and standardized data abstraction format between March 1^st^ and April 30^th^, 2025. RR was ascertained using the GeneXpert MTB/RIF assay or culture result as documented in patient records. Patients without documented drug susceptibility results were classified as drug-susceptible only if explicitly recorded as such.

**Study variables:** the dependent variable in this study was RR-TB. Whereas, the independent variables include sociodemographic factors (age, gender, residence, and living arrangement); clinical factors (close contact history with patients with TB, previous history of TB, site of infection, body mass index (BMI), TB type, GeneXpert MTB/RIF assay or culture-based drug susceptibility test result, and treatment category).

### Definitions

**Rifampicin-resistant tuberculosis (RR-TB):** tuberculosis with documented resistance to rifampicin detected by GeneXpert MTB/RIF assay or culture-based drug susceptibility testing as recorded in the patient´s medical record.

**History of TB:** a documented history of previous TB treatment prior to the current episode.

**Treatment category:** classification of patients with TB based on treatment history, as documented in the medical record. It includes: new, relapse, treatment failure, and transferred in [2].

**Body mass index (BMI):** body mass index recorded in the medical chart and calculated as weight in kilograms divided by height in meters squared (kg/m^^2^^), used to categorize patients as underweight, normal, or overweight [16].

**Statistical analysis:** the data were entered and cleaned using Kobo Toolbox, then exported to Stata version 17 for analysis. Descriptive statistics were applied to summarize the participants’ characteristics. A univariable logistic regression analysis was done to identify variables associated with RR-TB. Variables with a P-value < 0.25 were included in the multivariable logistic regression model to identify independent predictors of RR-TB. A P-value < 0.05 was deemed statistically significant, and adjusted odds ratios (aOR) with 95% confidence intervals (CI) were reported. Model fitness was checked using the Hosmer-Lemeshow test, and multicollinearity was evaluated using the variance inflation factor.

**Ethical considerations:** ethical clearance was granted by the Institutional Health Research Ethics Review Committee (IHRERC) of the College of Health and Medical Sciences, Haramaya University, Ethiopia (Ref. No: IHRER/056/2025). Permission to conduct the study was granted by Galkayo General Hospital, Puntland, Somalia. As the study involved retrospective record review, informed consent was obtained from the hospital administration. Confidentiality was maintained by anonymizing records and excluding personal identifiers throughout the investigation.

## Results

**Socio-demographic and clinical characteristics:** there were 422 patients with TB identified, but 410 of them who had complete records were included, yielding a response rate of 97.2%. The mean age was 29.9 (SD + 20.6) years, and male patients accounted for 64.6% of the total participants. Pulmonary TB represented 68.1% of the total cases, while the majority of the patients had no prior history of TB (78.8%) or exposure to a patient with TB (74.2%) (Table 1).

**Table 1:** socio-demographic and clinical characteristics of tuberculosis patients at Galkayo Hospital, Puntland, Somalia, 2020-2024

Variable	Category	n (%)
Age (years)	<15	99 (25.1)
	15-24	95 (23.2)
	25-34	68 (16.6)
	35-49	57 (13.9)
	≥50	91 (22.2)
Gender	Male	265 (64.6)
	Female	145 (35.4)
Residence	Urban	206 (50.2)
	Rural	204 (49.8)
Living arrangement	Alone	100 (24.4)
	With family	310 (75.6)
Close contact with a patient diagnosed with TB	No	304 (74.2)
	Yes	106 (25.8)
Previous history of TB	No	323 (78.8)
	Yes	87 (21.2)
Type of TB	Pulmonary	279 (68.1)
	Extrapulmonary	131 (31.9)
Treatment category	New	327 (79.8%)
	Relapse	39 (9.5%)
	Failure	11 (2.7%)
	Transferred in	33 (8%)
HIV status	Negative	407 (99.3%)
	Positive	3 (0.7%)
BMI	Underweight	132 (32.2)
	Normal	223 (54.4)
	Overweight	55 (13.4)

BMI: body mass index; TB: tuberculosis

**Prevalence of rifampicin resistance tuberculosis:** out of the 410 patients with TB analysed, 37 had RR-TB, yielding a prevalence of 9.02% (95% CI 6.43%-12.22%). Meanwhile, the prevalence among patients newly diagnosed with TB was 6.2% (20/323). The prevalence of RR-TB increased from 3.9% (3/77) in 2020 to 12.1% (11/91) in 2023; however, a slight decline was observed in 2024 (11.6%, 10/86) (Figure 1). Nevertheless, the overall difference across the five-year period was not statistically significant (χ^2^= 7.83, P=.10).

**Figure 1:**
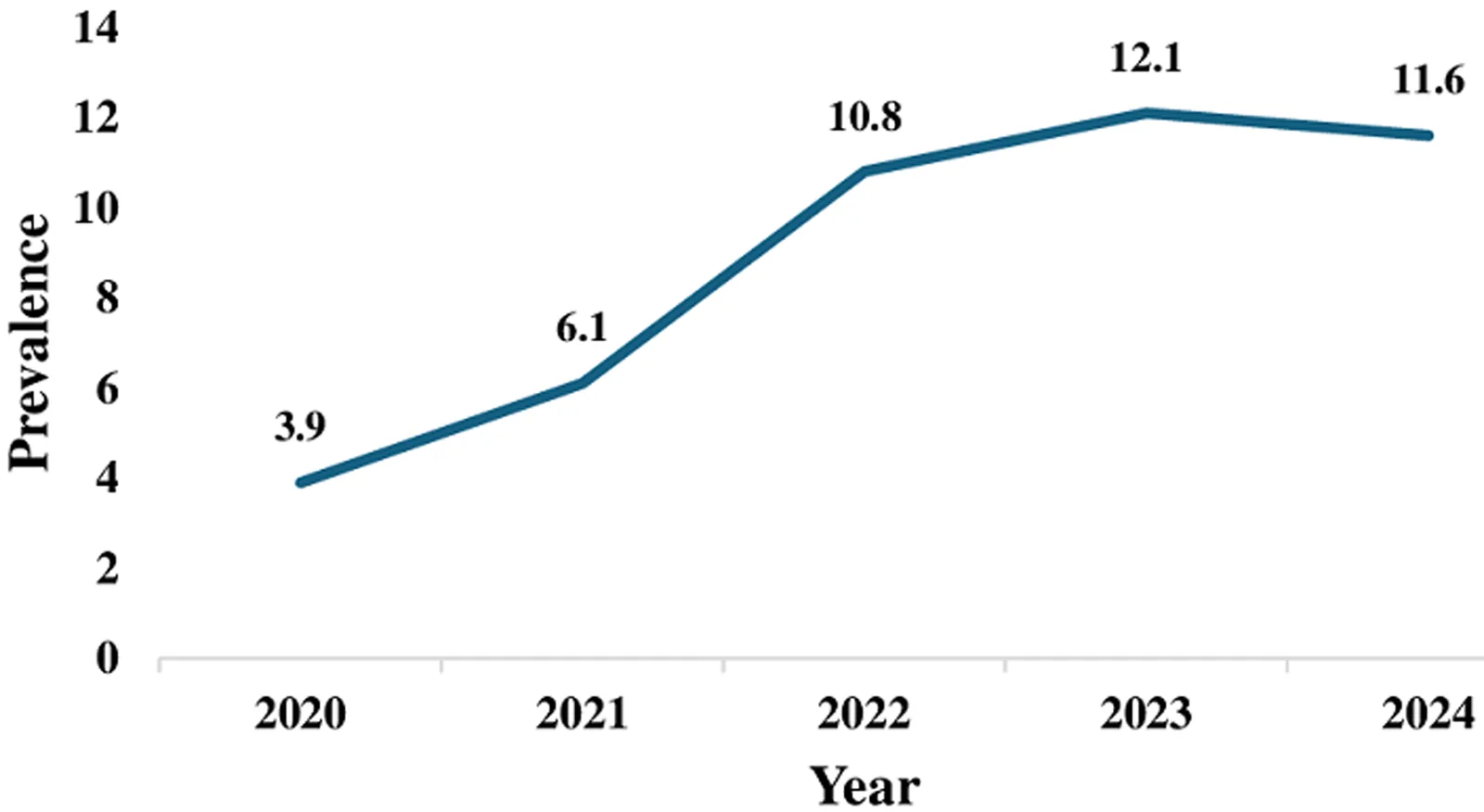
trend of RR-TB among patients with TB at Galkayo General Hospital, Puntland, Somalia, from 2020 to 2024

**Factors associated with rifampicin-resistant tuberculosis:** gender, residence, close contact history with a patient diagnosed with TB, previous TB history, treatment category, BMI, and living arrangement were included in the multivariable logistic regression model. In the final model, close contact with a patient with TB, previous history of TB, and treatment failure were significantly associated with RR-TB. Patients with a history of close contact had more than two times higher odds of RR-TB (aOR: 2.43, 95% CI: 1.13-5.21; P = 0.02). Those patients with previous history of TB, had more than three times higher odds of RR-TB (aOR: 3.63, 95% CI: 1.72-7.67; P <.001). Moreover, previous treatment failure was associated with RR-TB (aOR: 9.03, 95% CI: 2.03-40.12; P<.01) (Table 2).

**Table 2:** univariate and multivariable logistic regression analysis of variables associated with RR-TB among patients with TB attending Galkayo General Hospital, Puntland, Somalia, 2020-2024

Variables	Category	RR-TB		COR (95% CI)	P-value	AOR (95% CI)	P-value
		No	Yes				
Gender	Male	237	28	1.79 (0.82, 3.89)	0.15	1.74 (0.76, 3.98)	0.19
	Female	136	9	1		1	
Residence	Urban	182	24	1.94 (0.96, 3.92)	0.07	1.90 (0.89, 4.04)	0.10
	Rural	191	13	1		1	
Living arrangement	With family	279	31	1	0.23	1	
	Alone	94	6	0.57 (0.23, 1.42)		0.5 (0.19. 1.33)	0.17
Close contact with a patient diagnosed with TB	No Yes	282 91	22 15	1 2.11 (1.05, 4.24)	0.03	1 2.43 (1.13, 5.21)	0.02
Previous history of TB	No	303	20	1	<0.001	1	
	Yes	70	17	3.68 (1.83, 7.39)		3.63 (1.72, 7.67)	<0.001
Treatment category	New	302	25	1	;	1	
	Relapse	33	6	2.20 (0.84, 5.74)	0.11	1.95 (0.69, 5.46)	0.21
	Failure	7	4	6.90 (1.89, 25.19)	0.00	9.03 (2.03, 40.12)	<0.01
;	Transferred in	31	2	0.78 (0.18, 3.45)	0.74	0.80 (0.17, 3.83)	0.78
BMI (Kg/m^2^)	Under weight	116	16	1.67 (0.81, 3.43)	0.16	2.09 (0.94, 4.67)	0.07
	Normal	206	17	1		1	
	Overweight	51	4	0.95 (0.31, 2.95)	0.92	1.17 (0.35, 3.95)	0.80
Cigarette smoking	No	261	21	1	0.05	1	;
	Yes	102	16	2.02 (1.02, 4.03)		2.04 (0.96, 4.33)	0.06

RR-TB: rifampicin-resistant tuberculosis; BMI: body mass index; TB: tuberculosis; aOR: adjusted odds ratios

## Discussion

The study was conducted to determine the burden of RR-TB and its determinants among patients with TB at Galkayo General Hospital, Puntland, Somalia. The study found that the prevalence of RR-TB was 9.02%. Close contact with a patient with TB, previous history of TB, and treatment failure were identified as independent predictors of RR-TB. The observed prevalence of RR-TB in this study was 9.02%, indicating a considerable burden of drug-resistant TB in the setting. This finding is comparable with previous studies reported from Somalia (10.6%) [17] and Ethiopia (9.0%) [18], 9.3% [19], and 9.9% [20]. The observed prevalence is consistent with the global pooled prevalence of 9.4% reported in a recent meta-analysis [21]. However, the current prevalence is higher than reports from Kenya (4.6%) [22], South Africa (4.6%) [23], and Ethiopia (5.1%) [24]. These differences may reflect variations in TB control performance and a higher proportion of previously treated cases, a known risk factor for drug resistance [3,25]. Notably, the burden of RR-TB among newly diagnosed patients in this study was 6.2%, which is comparable to the aforementioned report from Ethiopia [24]. The relatively higher proportion of previously treated cases in our study (21%) versus the report from Ethiopia (7.2%) [24] may partly explain the overall burden observed. Nevertheless, the prevalence of RR-TB (6.2%) among newly diagnosed patients with TB remains concerning, as it suggests ongoing primary transmission of RR strains within the community.

Over the five years, the prevalence of RR-TB increased from 3.9% in 2020 to 12.1% in 2023, then declined to 11.6% in 2024. Although this pattern suggests a potential upward trend, the overall difference across the period was not statistically significant (χ^2^= 7.83, P = 0.098). A comparable result has been reported in a previous study conducted in Ethiopia [26]. Meanwhile, the lower prevalence observed in 2020 (3.9%) may partly reflect the impact of the COVID-19 pandemic on TB services. Globally, the pandemic disrupted routine TB screening, diagnostic services, and treatment follow-up, potentially leading to under-detection of RR-TB cases during 2020 [27-29]. The subsequent increase in RR-TB prevalence between 2021 and 2023 may be partly attributable to treatment interruptions and non-adherence, which is from restricted healthcare access, medicine shortages, and delayed diagnosis during the pandemic [30]. However, given that the overall trend was not statistically significant, these observations should be interpreted cautiously. The current study identified close contact history with a patient diagnosed with TB, previous TB treatment, and treatment failure as significant predictors of RR-TB. Patients with a history of close contact with a TB case had more than twice the odds of RR-TB. Similar associations have been reported in Ethiopia [31,32], and Burundi [33]. This relationship likely reflects primary transmission of resistant strains, particularly in household settings [34,35].

In Somalia, the impact may be fuelled by the living conditions of internally displaced and refugee populations. Hence, overcrowding, poor ventilation, and limited access to healthcare services may facilitate ongoing transmission of resistant TB [36,37]. Thus, the current study underscores the importance of strengthening contact tracing and community-based screening to reduce RR-TB. Previous history of TB was significantly associated with RR-TB, with affected patients having more than threefold increased odds. Similar findings have been reported in Somalia [14], Ethiopia [20], and Uganda [38]. Previous TB treatment is a well-established determinant of drug resistance [25]. Inadequate or incomplete therapy, poor adherence, inappropriate drug regimens, and substandard medications may promote the selection of resistant strains [39,40]. Hence, strengthening treatment adherence and close monitoring of previously treated patients is essential to prevent RR-TB.

Previous treatment failure was also strongly associated with RR-TB. Although the association was statistically significant, the wide CI indicates limited precision, possibly due to the small number of events in this category. Nevertheless, reports from similar studies conducted in Mogadishu, Somalia [14] and Ethiopia [4,1-4,3]. In this regard, treatment failure may reflect acquired resistance arising during therapy or previously undetected primary resistance [4,4]. Repeated treatment exposure further increases the likelihood of selecting resistant Mycobacterium TB strains [4,5]. In this regard, the current finding suggests the importance of routine drug susceptibility testing and close follow-up of patients to ensure timely detection and management of drug-resistant cases. This study is subject to the inherent limitations of retrospective secondary data analysis. Hence, incomplete or missing information on some variables. Moreover, the study was conducted in a single health facility, which may limit the generalizability of the findings to other regions of Somalia. Nevertheless, the study provides important evidence on the prevalence of RR-TB and its associated factors in the study setting where epidemiological data remain scarce.

## Conclusion

This study revealed a substantial burden of RR-TB in the study area. Previous TB treatment, treatment failure, and previous contact with a patient diagnosed with TB were significant predictors of RR-TB. These findings highlight the need for strengthened monitoring of patients with previous TB treatment and enhanced contact tracing strategies.

### What is known about this topic

- Rifampicin-resistant is a major challenge for global tuberculosis control;
- Previous tuberculosis treatment and treatment failure increase the risk of drug-resistant tuberculosis;
- Evidence on rifampicin-resistant from Somalia remains limited.

### What this study adds

- Rifampicin-resistant overall prevalence among patients with tuberculosis at Galkayo General Hospital was 9.02%;
- The prevalence of rifampicin-resistant among newly diagnosed patients with tuberculosis was 6.2% that suggests ongoing primary transmission of drug-resistant strains within the community;
- Close contact with a patient diagnosed with tuberculosis, previous history of tuberculosis, and treatment failure were independent predictors of RR-TB in this study.
